# Vegetations Lurking in the Dark and the Role of Neoendothelialization

**DOI:** 10.1016/j.case.2024.03.001

**Published:** 2024-04-06

**Authors:** Wissam Harmouch, Maha Yaqub, Servando Cuellar, Salman Salehin, Shahzad Ahmad, Mostafa Shalaby, Amer Abdulla

**Affiliations:** a Department of Internal Medicine, University of Texas Medical Branch, Galveston, Texas; b Division of Cardiovascular Medicine, University of Texas Medical Branch, Galveston, Texas

**Keywords:** Patent foramen ovale, Infective endocarditis, Transesophageal echocardiography, Congenital heart defect, Cryptogenic stroke

## Abstract

• IE of PFO devices is rare but devastating and necessitates surgical removal.
• Guidelines specify antibiotics for dental work, but epidural abscesses cause IE too.
• Incomplete NE of PFO devices is an independent risk factor for IE.
• Assessing NE after PFO closure may help individualize IE risk.
• Patients with intracardiac devices need counseling on dental and skin hygiene.

## Graphical abstract

**Figure:**
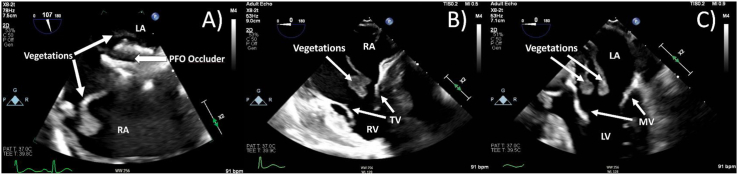

## Introduction

Percutaneous closure of an atrial septal defect (ASD) or patent foramen ovale (PFO) with an occlusion device has become the preferred treatment strategy compared to surgery. Percutaneous closure is safe and efficacious, results in shorter length of stay, and avoids a median sternotomy scar.^1^^,^^2^ Device complications include thromboembolic events, erosion, dislocation, valvular damage, atrial arrhythmias, and endocarditis.^3^ Atrial septal defect and PFO occlusion device-related endocarditis is an exceedingly rare complication, with very few case reports.^4^, ^5^, ^6^, ^7^ Typically, this complication occurs within 6 months of implantation due to incomplete endothelialization of the device with the native heart tissue. We present a unique case of infective endocarditis (IE) related to a PFO occluder device with large biatrial vegetations diagnosed 8 months after implantation.

## Case Presentation

A 59-year-old woman with a history of hypertension, former tobacco use, and a right lacunar cryptogenic stroke 8 months prior with subsequent elective PFO closure with a 25 mm device presented to the emergency department with severe lumbar back pain associated with paresthesias, lower extremity weakness, and urinary incontinence. The device was successfully implanted with no adjacent intracardiac leak identified on postoperative transthoracic echocardiography. The patient denied any recent falls or trauma. The patient was afebrile, had a blood pressure of 92/64 mm Hg, pulse of 58 beats per minute, respiratory rate of 14 breaths per minute, and oxygen saturation of 96% on room air. Examination revealed a small stage 1 pressure ulcer along the lumbar region with no other integumentary lesions. Neurologic exam revealed tenderness to palpation of the lumbar region and equally reduced lower extremity strength with active and passive motion bilaterally. Pertinent labs included a white blood cell count of 19.18 × 10^3^/μL, creatinine of 1.23 mg/dL, C-reactive protein of 36.2 mg/dL, and erythrocyte sedimentation rate of 120 mm/HR.

Magnetic resonance imaging with and without contrast of the lumbar spine revealed an epidural rim-enhancing lesion and enhancing collection from L3 to L4 measuring 0.8 × 0.6 × 3.7 cm with adjacent inflammation extending from L1 to L4. These findings suggested a large dorsal epidural abscess with surrounding phlegmon and compression of the cauda equina. The patient was urgently taken for hemilaminectomies and abscess evacuation.

Intraoperative tissue cultures yielded methicillin-sensitive *Staphylococcus aureus*, which was also present in both blood culture samples. Further history revealed that the patient did not have any prior epidural injections, peripheral intravenous lines, intravenous drug use, or recent dental or oral maxillofacial interventions. Blood cultures obtained 48 hours after starting appropriate antimicrobial therapy showed persistent bacteremia with methicillin-sensitive *Staphylococcus aureus*.

**Figure 1:**
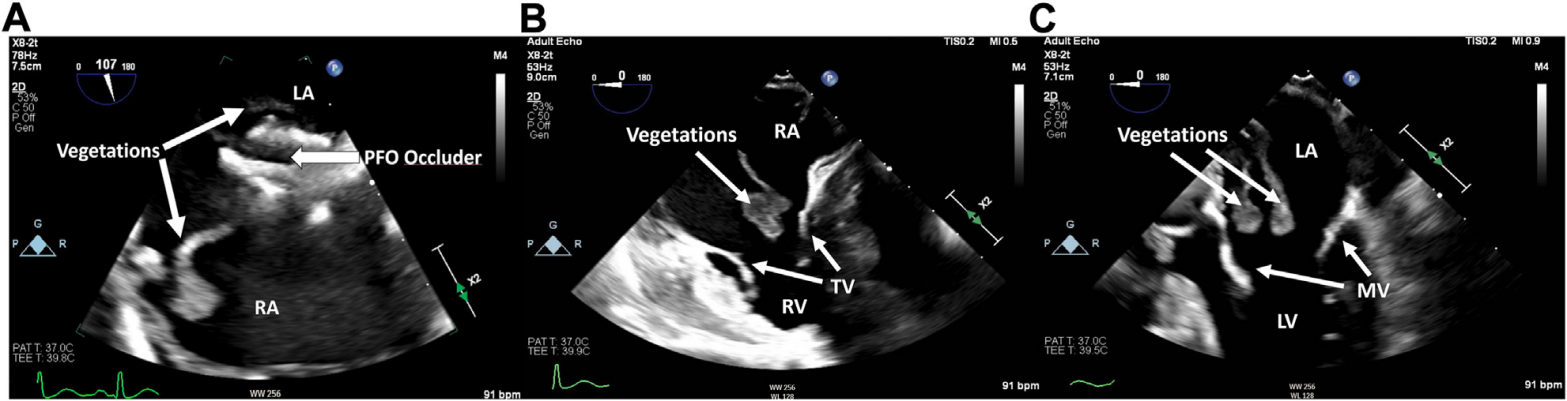
Two-dimensional transesophageal echocardiogram, midesophageal **(A)** bicaval, systolic view (107°), **(B)** modified, 4-chamber diastolic view (0°), and **(C)** 5-chamber diastolic view (0°), demonstrates the PFO occluder with large biatrial vegetations **(A)**, vegetations within the RA, but sparing the TV **(B)** and vegetations within the LA, but sparing the MV **(C)**. *LA*, Left atrium; *LV*, left ventricle; *MV*, mitral valve; *RA*, right atrium; *RV*, right ventricle; *TV*, tricuspid valve.

A transesophageal echocardiogram was obtained in the setting of persistent gram-positive bacteremia and concern for potential IE given the history of an implantable intracardiac device. Several large, mobile, heterogeneous, pedunculated masses were visualized attached to the PFO device in both atria (Video 1, Figure 1A). No vegetations were visualized on the native mitral or tricuspid valves (Figure 1B and C, Videos 2 and 3). The largest mass measured 3.0 cm in length.

A multidisciplinary discussion was held between cardiology, cardiothoracic surgery, and infectious disease. Cardiothoracic surgery removed the PFO device and surgically closed the PFO. When the right atrium was opened, several small vegetations were noted attached to the PFO closure device and removed. The device was freed up bluntly and as atraumatically as possible. The extent of neoendothelialization (NE) could not be determined. The tissue was sent for pathological analysis and described fibrin with acute inflammation, consistent with vegetation. Gram stain and fungal cultures were negative. The patient had an uneventful postoperative course and was discharged with intravenous cefazolin and oral rifampin for 6 weeks of duration. At follow-up, they completed the course of antibiotics and continued to have improved functional capacity with no neurologic deficits or cardiac complications.

## Discussion

The American Heart Association (AHA) assigns a class 2a recommendation for PFO closure for patients 18 to 60 years of age for secondary prevention of stroke if high-risk anatomic features, such as large shunt size and atrial septal aneurysm, are present.^8^ These guidelines come from the DEFENSE-PFO and RESPECT trials that showed an improved mortality benefit for PFO closure compared to medical management alone in these high-risk patients.^9^^,^^10^ As PFO closure devices have become more favorable over the years, the potential complications of device implantation have become more evident. Specifically, IE is a rare phenomenon that can occur with ASD and PFO closure devices. The 2018 American College of Cardiology/AHA congenital heart disease guidelines recommend patients with PFO closure devices undergoing invasive dental or oral procedures receive antibiotic prophylaxis within 6 months of implantation of the device or if there is a residual intracardiac shunt adjacent to the device.^11^ Our patient required surgical removal of the infected closure device followed by surgical PFO closure.

This complication has been documented in all age groups and patient populations, including infants, pregnant women, adult males, and elderly patients. These patients had various clinical presentations that included systemic symptoms, including persistent fever, impaired general condition, embolic phenomenon, petechiae, and septic shock.^7^ Our patient uniquely presented with neurological symptoms, including severe back pain with associated paresthesias, lower extremity weakness, and urinary incontinence. This presentation was unique in the sense of a complicated skin and soft tissue infection that led to cauda equina syndrome and subsequent spread of the infection to the blood stream where the bacteria were able to seed the PFO device and lead to IE. One case from the literature described a 73-year-old woman who developed IE 6 years after device implantation and was found to have incomplete NE of the device from pathology examination.^12^ The suggested inciting event occurred 2 months prior to presentation when the patient had undergone a dental procedure without antibiotic prophylaxis. Interestingly, our patient had no recent dental procedures prior to presentation. The precipitating factor was thought to be a pressure wound with progression to epidural abscess. There have been a few other cases of IE reported in the literature that cite incomplete NE upon device removal and IE despite antimicrobial prophylaxis.^7^ Infective endocarditis of these devices occurred days and even years after initial placement. These cases challenge the notion that NE occurs by 6 months of device implantation. As current AHA guidelines recommend antibiotic prophylaxis for IE prevention in patients who undergo a dental procedure with a prosthetic device placed within 6 months, these reported cases suggest that longer-term antibiotic prophylaxis may be warranted in some patients.

On transesophageal echocardiography, our patient was found to have several large, mobile, pedunculated vegetations on both the left and right atrial surfaces of the PFO closure device, with the largest vegetation measuring up to 3 cm. Large vegetations greater than 1 cm have increased risk of embolization and mortality.^13^ No visible shunt was noted via color-flow Doppler. Upon surgical removal, vegetations were visibly attached to the device. The histopathology was consistent with vegetations. The extent of NE could not be determined. Microbiology analysis was overall negative. While the mechanism of device IE is likely multifactorial, incomplete NE should be considered a major risk factor. Furthermore, there have been 25 reported cases of IE after device closure of ASDs.^4^, ^5^, ^6^, ^7^^,^^12^ Sixteen of these patients developed IE more than 6 months after device implantation and can be classified as late IE. Eight of those with late IE had incomplete or absent NE on device explant. Additionally, given the occurrence of IE despite NE in some instances, the exact role of this phenomenon is not fully understood in association with the risk of IE. Future studies are needed to more fully understand the association of NE and the potential for IE in patients with PFO device implants. However, patients may benefit from imaging to determine the degree of NE in vivo so that management can be individualized. Angioscopic assessment of PFO occluder device NE has been reported.^14^ Additionally, a recent retrospective study suggested newly developed radiological criteria, using cardiac computed tomography (CCT), to assess NE 6 months after PFO device implantation.^15^ The study used CCT imaging to assess device size and opacification. The central thickness of the device was generally 3 to 4 mm at implantation. A device with a central thickness ≤6 mm was considered flat, and devices with a central thickness >6 mm were considered bulky. In conjunction with device shape, device contrast opacification was also assessed. Contrast enhancement of the entire device was considered fully opacified. Any visible diffusion of contrast through the atrial surface of the device was categorized as partially opacified. If there was no contrast opacification within the device and the shape was flat, then NE was considered complete. These findings challenge the current recommendations for a standard 6-month duration of antibiotic prophylaxis for patients and suggest the need to assess the degree of NE through imaging.

Despite adherence to current guidelines, our patient developed PFO device IE, even in the absence of a recent dental procedure. Such case reports call for further studies to help develop measures that prevent this complication. For example, certain high-risk patients may benefit from indefinite antibiotic prophylaxis, and perhaps routine angioscopy or CCT to assess NE to better inform risk of IE and individualize management. Additionally, patients should be counseled on the importance of proper skin protection, such as the utilization of gloves and skin barrier protection, as our case suggests our patient's IE may have been caused by a soft tissue infection and epidural abscess.

## Conclusion

Infective endocarditis is a rare but serious complication of percutaneous PFO closure. Current guidelines recommend antibiotic prophylaxis prior to invasive dental or oral procedures during the 6 months following PFO closure with the assumption that complete NE should have occurred after 6 months and that risk of IE should therefore be low. There have been several PFO closure-related IE cases that occurred beyond 6 months, including our patient. This emphasizes the importance of ruling out a residual shunt adjacent to the prosthetic material on postoperative echocardiography, as this would warrant indefinite antibiotic prophylaxis. It also may suggest the need to develop and implement imaging techniques to assess the extent of NE and consider recommending longer-term antibiotic prophylaxis on a personalized, image-guided basis.

## Ethics Statement

The authors declare that the work described has been carried out in accordance with The Code of Ethics of the World Medical Association (Declaration of Helsinki) for experiments involving humans.

## Consent Statement

The authors declare that since this was a noninterventional, retrospective, observational study utilizing de-identified data, informed consent was not required from the patient under an IRB exemption status.

## Funding Statement

The authors declare that this report did not receive any specific grant from funding agencies in the public, commercial, or not-for-profit sectors.

## Disclosure Statement

The authors report no conflict of interest.

## Supplementary Data

Video 1 Two-dimensional transesophageal echocardiogram (TEE), midesophageal bicaval view (107°) without (*left*) and with (*right*) color-flow Doppler, demonstrates the PFO device with large pedunculated vegetations attached to the right and left atrial sides of the device without atrial level shunting.

Video 2 Two-dimensional TEE, midesophageal modified midesophageal 4-chamber view (0°), focused on the tricuspid valve, demonstrates the vegetations within the right atrium, but sparing the tricuspid valve.

Video 3 Two-dimensional TEE, midesophageal 5-chamber view (28°) without (*left*) and with (*right*) color-flow Doppler, demonstrates the vegetations within the left atrium, but sparing the mitral valve; mild mitral regurgitation is also demonstrated.
